# Predictors of Improved Walking after a Supervised Walking Exercise Program in Men and Women with Peripheral Artery Disease

**DOI:** 10.1155/2016/2191350

**Published:** 2016-12-25

**Authors:** Andrew W. Gardner, Donald E. Parker, Polly S. Montgomery

**Affiliations:** ^1^Reynolds Oklahoma Center on Aging, Donald W. Reynolds Department of Geriatric Medicine, University of Oklahoma Health Sciences Center (OUHSC), Oklahoma City, OK, USA; ^2^Veterans Affairs Medical Center, Oklahoma City, OK, USA; ^3^Department of Biostatistics and Epidemiology, OUHSC, Oklahoma City, OK, USA

## Abstract

We compared the changes in ambulatory outcomes between men and women with symptomatic peripheral arterial disease (PAD) following completion of a supervised, on-site, treadmill exercise program, and we determined whether exercise training variables and baseline clinical characteristics were predictive of changes in ambulatory outcomes in men and women. Twenty-three men and 25 women completed the supervised exercise program, consisting of intermittent walking to mild-to-moderate claudication pain for three months. Men and women significantly increased claudication onset time (COT) (*p* < 0.001 and *p* < 0.01, resp.) and peak walking time (PWT) (*p* < 0.001 for each group). However, change in PWT was less in women (54%) than in men (77%) (*p* < 0.05). Neither group significantly changed 6-minute walk distance (6MWD). In women, baseline COT was the only predictor for the change in COT (*p* = 0.007) and the change in PWT (*p* = 0.094). In men, baseline COT (*p* < 0.01) and obesity (*p* < 0.10) were predictors for the change in COT, and obesity was the only predictor for the change in PWT (*p* = 0.002). Following a supervised, on-site, treadmill exercise program, women had less improvement in PWT than men, and neither men nor women improved submaximal, overground 6MWD. Furthermore, obese men and patients with lower baseline COT were least responsive to supervised exercise. This trial is registered with ClinicalTrial.gov, unique identifier: NCT00618670.

## 1. Introduction

Peripheral artery disease (PAD) is a highly prevalent [1], costly [2], and deadly condition [3]. Additionally, PAD results in low patient-perceived health-related quality of life, primarily due to ambulatory leg pain and dysfunction [4]. Consequently, patients with PAD have impaired physical function [5], low physical activity levels [6], and faster rates of functional decline and mobility loss over time compared to those without PAD [5].

Supervised exercise therapy is highly efficacious in treating symptomatic PAD and has been given a Class IA recommendation by the American College of Cardiology and the American Heart Association [1]. Although exercise rehabilitation improves claudication, ambulation, physical function, and quality of life [7–10], it is less clear whether the volume of exercise completed during a program of supervised exercise is predictive of a more favorable response in both men and women with PAD. We previously found that exercise-mediated improvements in claudication onset time (COT) and peak walking time (PWT) occur rapidly within the first two months of exercise rehabilitation, but this was primarily in men [11]. A recent meta-analysis found that exercise training variables did not predict change in COT and PWT, but this study did not evaluate whether sex-specific differences existed in exercise training variables, and the analysis was conducted at the study level, which does not take into consideration individual patient differences in completing exercise programs [12]. From an exercise standpoint, information about the components of an exercise program is important to tailor a more individualized exercise prescription to different subgroups of patients.

In addition to considering the components of an exercise program, baseline characteristics such as age, body mass index, baseline PWT, cardiac comorbidities, smoking, pulmonary disease, and sex have been found to predict response to exercise [13, 14]. We have previously shown that numerous outcome measures are different between men and women with PAD, as women have greater impairment in baseline ambulation [15], vascular function [16], and inflammation [17]. Furthermore, women, particularly those with diabetes, have less increase in COT and PWT following supervised exercise training [18, 19], even though their increase in 6-minute walk distance (6MWD) following a home-based exercise program was similar [10]. Relatively little is known about whether exercise training variables in combination with baseline measures are predictive of improvements in claudication outcomes following a program of supervised exercise in patients with PAD and whether predictors are sex-specific. From a clinical standpoint, it is important to determine whether men and women who are more responsive to exercise can be identified.

To address these issues, we performed a follow-up, exploratory analysis within the supervised exercise group from our recent prospective, randomized controlled exercise trial in symptomatic patients with PAD [8]. Our specific aims were to compare the changes in ambulatory outcomes between men and women with symptomatic PAD following completion of a supervised, on-site, treadmill exercise program and to determine whether exercise training variables in combination with baseline clinical characteristics were predictive of changes in the ambulatory outcomes in men and women.

## 2. Methods

### 2.1. Patients

#### 2.1.1. Approval and Informed Consent

The institutional review board at the University of Oklahoma Health Sciences Center (HSC) and the Research and Development committee at the Oklahoma City VA Medical Center approved the procedures of this study. Written informed consent was obtained from each patient at the beginning of the investigation.

#### 2.1.2. Recruitment

Patients were recruited from vascular labs and vascular clinics from the University of Oklahoma HSC and the Oklahoma City VA Medical Center.

### 2.2. Baseline Clinical Characteristics Obtained from a Medical History and Physical Examination

Patients were evaluated in the morning at the Clinical Research Center, at the University of Oklahoma HSC. Patients arrived fasted but were permitted to take their usual medications. To begin the study visit, patients were evaluated with a medical history and physical examination in which demographic information, height, mass, waist circumference, cardiovascular risk factors, comorbid conditions, claudication history, ankle/brachial index (ABI), blood samples, and a list of current medications were obtained. Obesity was defined by a body mass index ≥30, and abdominal obesity was defined by a waist circumference ≥102 cm in men and ≥94 cm in women [20]. Hypertension was defined by having at least one of the following conditions: a systolic blood pressure ≥140 mmHg, a diastolic blood pressure ≥90 mmHg, or currently taking antihypertensive medications. Dyslipidemia was defined by having at least one of the following conditions: a cholesterol value ≥200 mg/dL, a triglyceride value ≥150 mg/dL, a low-density lipoprotein level ≥130 mg/dL, a high-density lipoprotein level <40 mg/dL in men and <50 mg/dL in women, or currently taking lipid-lowering medications. Diabetes was defined by having at least one of the following conditions: a fasting blood glucose ≥126 mg/dL or currently taking oral medication or insulin. Metabolic syndrome was defined according to the National Cholesterol Education Program (NCEP) Adult Treatment Panel (ATP) III [20], as having three or more of the following criteria: (1) abdominal obesity (waist circumference >102 cm in men and >88 cm in women), (2) hypertriglyceridemia (≥150 mg/dL), (3) low HDL cholesterol (<40 mg/dL in men and <50 mg/dL in women), (4) blood pressure ≥130/85 mmHg, and (5) fasting glucose ≥110 mg/dL. Although several definitions of metabolic syndrome exist [20, 21], the NCEP ATP III definition was used in this investigation because it was specifically established on a population from the United States. A history of lower extremity revascularization, smoking, cerebral vascular accidents (stroke or transischemic attacks), and prior angina was determined by self-report, and a history of COPD was determined if the participants reported that they had been diagnosed with COPD by their physician.

#### 2.2.1. Inclusion and Exclusion Criteria

Patients with symptomatic PAD were included in this study if they met the following criteria: (a) a history of ambulatory leg pain, (b) ambulatory leg pain confirmed by treadmill exercise [4], and (c) an ABI ≤0.90 [1] at rest or ≤0.73 after exercise [22]. Patients were excluded for the following conditions: (a) absence of PAD (ABI > 0.90 at rest and ABI > 0.73 after exercise), (b) noncompressible vessels (ABI ≥ 1.40), (c) asymptomatic PAD, (d) use of medications indicated for the treatment of claudication (cilostazol or pentoxifylline) initiated within three months prior to investigation, (e) exercise currently limited by other diseases or conditions, but a previous history of a disease or condition (e.g., angina) was not exclusionary, (f) active cancer, (g) end stage renal disease defined as stage 5 chronic kidney disease, (h) abnormal liver function, (i) failure to complete all of the baseline tests in a 3-week baseline run-in phase, and (j) failure to complete more than one-third (>12) of the exercise intervention sessions. A total of 60 patients were enrolled in the supervised exercise program, of which eight patients did not complete the study and four others completed only 12 or fewer of the prescribed 36 exercise sessions. Thus, 48 patients who successfully completed the study were included in the analyses. Unlike in our previous randomized controlled trial in which we performed intent-to-treat analyses to assess efficacy of exercise, the primary point of this follow-up paper is to determine the changes that occur in those patients who actually adhere to and complete the supervised exercise program.

### 2.3. Supervised Exercise Rehabilitation Program

Exercise sessions in our supervised, on-site, treadmill exercise program were performed while wearing a small (75 × 50 × 20 mm), lightweight (38 g) step activity monitor (StepWatch3™, Orthoinnovations, Inc., Oklahoma City, OK), as previously described [7, 8]. Briefly, the supervised program consisted of three months of intermittent treadmill walking to mild-to-moderate claudication pain three days per week at a speed of approximately two mph and at a grade equal to 40% of the highest work load achieved during the baseline maximal treadmill test. Exercise sessions progressively increased during the program from 15 to 40 minutes. This program increases the volume of exercise via progressive increments in duration rather than intensity because we have previously shown that a lower-intensity program elicited similar ambulatory improvements as compared to a higher-intensity program [23]. To quantify volume of exercise performed, expressed as MET-minutes, intensity of each exercise training session was determined by using the oxygen uptake value obtained during the baseline maximal treadmill test that corresponded to the exercise training grade, and this MET value was multiplied by the duration to yield MET-minutes as previously described [7, 8].

### 2.4. Primary Outcome Measures

#### 2.4.1. COT and PWT Obtained from a Graded Maximal Treadmill Test

Patients performed a graded treadmill test (2 mph, 0% grade with 2% increase every 2 minutes until maximal claudication pain) to determine study eligibility and then repeated the test on a following baseline visit within one week and again after the 3-month exercise program to obtain the primary outcome measures of COT and PWT as previously described [4, 7]. We prefer to report these claudication outcomes in units of time because the work stages during the treadmill test increase every 2 minutes, thereby making the work stages associated with the occurrence of COT and PWT simple to determine. Given that the treadmill test was performed at a constant speed of 2 mph, the conversion of COT and PWT to distances can be done by converting the walking speed from 2 mph to 53.6 meters per second. Using our procedures, the test-retest intraclass reliability coefficient is *R* = 0.89 for COT [4] and *R* = 0.93 for PWT [4].

#### 2.4.2. Total Walk Distance Obtained from a 6-Minute Walk Test

On a separate day, typically within one week from the final treadmill test, patients performed an overground, 6-minute walk test supervised by trained exercise technicians, as previously described from our laboratory [24]. The total distance walked during the test was recorded. The test-retest intraclass reliability coefficient is *R* = 0.94 for total 6-minute walking distance [24].

### 2.5. Statistical Analyses

In initial analysis, large differences between men and women were observed in the linear associations among the change scores and baseline variables. While a model with sex related interactions was an option, we chose the equally valid approach of separate models and data summaries for men and women. This has the advantage of a simpler model, requires less statistical assumptions, and emphasizes the combination of variables most important to each sex.

The baseline treadmill variables exhibited significant skewing and were summarized within each sex as medians and interquartile ranges. Wilcoxon tests were used for testing these variables. Other measurement variables at baseline were summarized within each sex as means and standard deviations, and independent *t*-tests were used for testing among pairs of means. Dichotomous variables were summarized as percent with attribute present and were compared between sexes using a single degree of freedom chi-squared test. Similar tests of baseline variables between subjects who completed the exercise program and those who did not complete revealed no significant differences. Exercise intervention measures were compared within each sex group as the time simple effects of a 2-time-by-2 group ANOVA with repeated measure on time. The interaction term was used to compare differences in simple effects across groups. Note that these tests assume normality only for the change scores and not for the baseline and posttest measures as would be required for main effects tests. To provide information regarding the clinical relevance of the change scores, we calculated the effect size for COT and PWT in men and women, defined as the mean change score/standard deviation of the change score.

Multivariate regression predictor models for values of outcome variables after completion of the supervised exercise program were obtained using an iterative least square procedure with baseline variables and exercise training variables as possible predictors. The variable producing the largest *R*-square value was entered first and variable producing the largest change in *R*-square entered in each subsequent step. To avoid overparameterization, a maximum of three variables were allowed in the model and were restricted to only those with *p* value <0.10. Associations among pairs of variables were measured using correlations. NCSS computer package (Kaysville, UT) was used for all computations.

## 3. Results

### 3.1. Baseline Clinical Characteristics

The clinical characteristics of the men and women are shown in Table 1. The groups had mean ABI values typical for symptomatic PAD and consisted of a mix of older, overweight, and obese Caucasians and African-Americans with a high prevalence of cardiovascular risk factors and comorbid conditions. The women had significantly higher prevalence of diabetes (*p* < 0.05) than the men.

### 3.2. Exercise Training and Ambulatory Measures

Exercise training and ambulatory measures are shown in Table 2. On average, the men and women completed 89% and 92% of the possible 36 exercise training sessions, respectively. None of the training variables, expressed either as the total amount performed during the program or as the average amount performed per exercise session, were significantly different between the men and women. At baseline, women had a significantly lower 6MWD than men (*p* < 0.05). Men and women significantly increased COT (*p* < 0.001 and *p* < 0.01, resp.) and PWT (*p* < 0.001 for each group). However, the change in PWT was less in the women (54%) than in the men (77%) (*p* < 0.05). Neither group significantly changed 6MWD. Consequently, correlates and predictors of changes scores were only determined for COT and PWT.

### 3.3. Correlates of the Changes in COT and PWT

The strongest univariate correlates of the change in ambulatory outcomes are displayed in Table 3. In women, the baseline COT (*p* < 0.01) and PWT (*p* < 0.01) were the highest univariate correlates (positive) with the change in COT, and baseline COT (*p* < 0.10) was also the highest correlate with the change in PWT. In men, the baseline COT (*p* < 0.001), PWT (*p* < 0.001), and 6MWD (*p* < 0.01) and total volume of exercise (*p* < 0.01) were the highest univariate correlates (positive) with the change in COT. Obesity (*p* < 0.01), abdominal obesity (*p* < 0.01), BMI (*p* < 0.01), and history of lower extremity revascularization (*p* < 0.05) were the highest correlates (negative) with the change in PWT.

### 3.4. Multivariate Regression Predictors of Changes in COT and PWT

In women, the multivariate regression models identifying predictors of the changes in ambulatory outcomes are presented in Table 4. The baseline COT was the only predictor that entered the model for the change in COT (*p* = 0.007) and the change in PWT (*p* = 0.094). In men (Table 5), baseline COT (*p* < 0.01) and obesity (*p* < 0.10) were the predictors that entered the model for the change in COT, and obesity was the only predictor that entered the model for the change in PWT (*p* = 0.002).

## 4. Discussion

The primary findings were that (a) both men and women increased COT and PWT following the supervised, on-site, treadmill exercise program, but 6MWD did not change in either group, (b) women had a smaller absolute and relative increase in PWT than men, (c) obesity was a significant predictor for the change in COT and PWT in men, but not in women, and (d) baseline COT was a significant predictor for the change in COT and PWT in both men and women.

### 4.1. Exercise Adherence to Supervised Exercise in Men and Women

The adherence to the supervised, on-site exercise program was high in the patients, as the men and women completed 89% and 92% of the possible 36 exercise sessions in the program, respectively. Furthermore, the men and women completed a similar amount of exercise during the exercise program, as measured by the mean exercise time, strides, cadence, and MET-min completed per exercise session. The similarity of exercise training variables in this study compares favorably to our recent report comparing men and women who completed a home-based exercise program [19]. The only difference between our two studies is that the women in the current investigation walked at a mean cadence of 46 strides per minute during the supervised treadmill training, which was not different than the men. In contrast, the women in our previous study walked at a mean cadence of only 35 strides per minute during the home-based exercise program, which was significantly slower than the men. The exercise training results in the current study indicates that the men and women received a similar exercise dose during the 12-week intervention. Although different modes of supervised exercise programs could have been selected for the training program to improve claudication outcomes, such as resistance training [25, 26], we chose to utilize a supervised treadmill walking program as described in our methods because walking is the specific activity which elicits symptoms, and we have found that supervised walking programs are efficacious to improve claudication outcomes [7, 8].

### 4.2. Efficacy of Supervised Exercise in Men and Women

This is the first study to directly compare the exercise training dose and the changes in COT, PWT, and 6MWD in men and women following a supervised exercise program. Despite completing a similar dose of exercise during the program, women had a 47% smaller increase in PWT than the men. Women increased their PWT by more than two minutes, whereas men increased their PWT by more than four minutes. Given that the treadmill test protocol increases grade in two-minute increments, the sex difference in the change in PWT has additional physiological significance, as the men increased their exercise capacity by more than 0.55 MET's higher than the women.

The supervised exercise program resulted in significant and very large clinically meaningful improvements in COT and PWT in both men and women. In men, the effect size was 1.13 for the change score of both COT (213 seconds/188 seconds; see Table 2) and PWT (265 seconds/235 seconds), far surpassing the criteria of an effect size of 0.5 which defines a large clinically meaningful change [27]. In women, the effect size was 0.73 for the change score of COT and was 0.88 for the change score of PWT, both of which are very large and clinically meaningful changes [27]. These findings support previous work reporting improvements in COT and PWT following supervised exercise training [7–9]. These results indicate that a supervised exercise program is an efficient intervention to improve claudication pain and exercise capacity in symptomatic patients with PAD,

The supervised exercise program did not elicit significant improvements in 6MWD in either men or women. However, the effect size for the change score in 6MWD was 0.36 in men and 0.22 in women, both of which surpass the criterion of an effect size of 0.2 which defines a small clinically meaningful change [27]. The lack of statistical significance was most likely due to the small sample sizes to detect small effect size changes. The lack of significant improvement in 6MWD in the men and women in the current study does not support previous studies from our lab and others which found significant increases with larger samples sizes [8, 10]. However, the smaller change score effect sizes for 6MWD compared to those found for COT and PWT illustrate the specificity of exercise training. Supervised treadmill training elicits greater improvements in treadmill outcomes (COT and PWT) compared to an overground walking outcome such as 6MWD.

### 4.3. Predictors of Changes in Ambulatory Outcomes

This is the first study to examine whether the combination of exercise training measurements and baseline characteristics can be used to predict change in COT and PWT following a supervised exercise program in men and women separately. In women, the baseline COT value was positively associated with the change scores of COT and PWT and was the only predictor to enter the models. Thus, women who had higher COT at baseline had greater increases in COT and PWT in response to the supervised exercise program. This finding suggests that women with more severe claudication at baseline may need either a greater dose of exercise in the supervised program, or an additional intervention combined with treadmill walking, or both to elicit more optimal improvements in COT and PWT. In a recent report we found that baseline measures in women were not predictive of the change scores in COT, PWT, and 6MWD following a home-based exercise program [19], indicating that women with more severe claudication responded as well as women with less severe symptoms. Therefore, women who experience COT quickly at baseline may further benefit from a home-based exercise program used in combination with a supervised exercise program or by itself, provided that the walking is done at a relatively fast cadence.

Similar to the women, the baseline COT value of the men was positively associated with the change score of COT and was one of two predictors to enter the model. Therefore, men who had higher baseline COT had a greater increase in COT in response to the exercise program. Unlike the women, however, obesity was negatively associated with the change scores of both COT and PWT and was a predictor that entered the models. These findings indicate that men who were obese at baseline had smaller increases in COT and PWT following the supervised exercise program, which is supported by our recent study that found men who had metabolic syndrome had less increase in COT following a home-based exercise program [19]. Thus, in men the combination of having low baseline COT and obesity was particularly detrimental to increase COT with a supervised exercise program, and having obesity by itself limited the improvement in PWT. These findings regarding the negative consequences of obesity on symptoms are supported by previous work in our laboratory [28] and by others [29, 30]. We have reported that body mass index is an independent predictor negatively associated with baseline COT and PWT in symptomatic patients with PAD [31]. Furthermore, we have shown that PAD patients with metabolic syndrome have lower COT, PWT, 6MWD, health-related quality of life, and perceived walking ability and that abdominal obesity was the primary component of metabolic syndrome related to poorer exercise performance [28]. Others have found that obesity is strongly associated with worse claudication symptoms in patients with PAD [32], lower baseline COT [29], and greater rates of decline in 6MWD and gait speed during a four-year follow-up period [30].

### 4.4. Limitations

Although this trial supports the efficacy of a supervised exercise program for men and women with PAD and identifies predictors of the changes in ambulatory outcomes, several limitations exist. One limitation is that patients volunteered to participate in this study. Thus, a self-selection bias may exist because they may represent those who had greater interest in participating, better access to transportation to the research center, and better health than PAD patients who did not volunteer. A second limitation is that the results of this study are only generalizable to patients with symptomatic PAD and may not be applicable to patients with less severe or more severe PAD. Another limitation is that, by definition, we chose to study only those who successfully adhered to and completed the exercise program (48 out of 60) because we were interested in describing the true potential of the supervised exercise program to change ambulatory outcome measures if patients successfully adhered to the program. We have already reported the efficacy of supervised exercise programs using several methods to rigorously account for bias due to dropping out of the study or poorly attending the program [7, 8]. These methods included using an intent-to-treat analytic strategy, which analyzed those patients who completed the study but who had low adherence rates to the intervention, and we compared the baseline characteristics of patients who completed with those who did not complete. We believe there is minimal bias associated with failing to complete or poorly attending the program because similar results were obtained using intent-to-treat versus completer analyses and because the baseline characteristics of drop outs were not different than those who completed the study [7, 8]. A final limitation is that the sample size was small, which limits the ability to identify significant correlates of the change scores for COT and PWT, and it provides less precise estimates of change scores than if a larger sample were available. However, it should be noted that, even with a small sample size, COT and PWT change scores in men and women were significant due to the large effect sizes associated with the supervised exercise program and the fact that predictors of the change scores were identified in multivariate models.

## 5. Conclusion and Clinical Significance

We conclude that, following a supervised, on-site, treadmill exercise program, women had less improvement in PWT than men, and neither men nor women improved submaximal, overground 6MWD. Furthermore, obese men and patients with lower baseline COT were least responsive to supervised exercise. The clinical significance is that a greater dose of exercise and/or a concomitant intervention may be needed for more optimal improvements in women, obese men, and those with poor baseline ambulation, particularly if the desired outcome is to increase 6MWD.

## Figures and Tables

**Table 1:** Baseline clinical characteristics of men and women with symptomatic peripheral artery disease who completed the supervised exercise program.

Variables	Men (*N* = 23)	Women (*N* = 25)
*Means (SD)*		
Age (y)	66 (10)	63 (11)
Ankle/brachial index	0.66 (0.26)	0.69 (0.24)
Mass (kg)	85.0 (21.2)	78.3 (17.6)
Body mass index (kg/m^2^)	28.7 (7.0)	30.1 (7.2)
*Percentage with characteristics present*		
Race (% Caucasian)	65	40
Current smoking (% yes)	30	48
Hypertension (% yes)	83	92
Dyslipidemia (% yes)	74	76
Diabetes (% yes)	35	64^*∗*^
Obesity (% yes)	39	52
Abdominal obesity (% yes)	39	64
Metabolic syndrome (% yes)	78	88
Lower extremity revascularization (% yes)	28	35
Previous history of angina (% yes)	26	24
Cerebral vascular accident (% yes)	17	12
Chronic obstructive pulmonary disease (% yes)	30	36

^*∗*^Different than men (*p* < 0.05).

**Table 2:** Exercise measures and ambulatory outcome change scores. Values are means (SD) unless otherwise specified.

Variables	Men (*N* = 23)	Women (*N* = 25)
*Exercise training measures*		
Exercise sessions completed (*n*)	32 (7)	33 (5)
Average exercise time (min/exercise session)	26.1 (3.0)	25.9 (2.8)
Average exercise strides (strides/exercise session)	1116 (172)	1196 (229)
Average exercise cadence (strides/min)	42.9 (5.0)	46.0 (6.8)
Total volume of exercise (MET-min)	2394 (691)	2484 (730)
*Baseline ambulatory measures*		
Claudication onset time (s)^§^	171 (121)	136 (*102*)
Peak walking time (s)^§^	345 (310)	263 (228)
6-minute walk distance (m)	344 (92)	308 (76)^*∗*^
*Change scores of ambulatory outcome measures (posttest value − baseline value)*		
Change in claudication onset time (s)	213 (188)^‡^	145 (199)^†^
Change in peak walking time (s)	265 (235)^‡^	141 (160)^‡*∗*^
Change in 6-minute walk distance (m)	22 (61)	8 (36)

^*∗*^Different than men (*p* < 0.05).

^†^Change different from zero (*p* < 0.01) and ^‡^(*p* < 0.001).

^§^Values are medians (interquartile range).

**Table 3:** Four strongest univariate correlates of the change in ambulatory outcomes with baseline characteristics in symptomatic patients with peripheral artery disease.

Group	ΔCOT	ΔPWT
Women	Baseline COT (*r* = 0.52)^†^	Baseline COT (*r* = 0.35)
	Baseline PWT (*r* = 0.52)^†^	Angina (*r* = −0.31)
	Previous history of angina (*r* = −0.36)	Body mass index (*r* = −0.22)
	Lower extremity revascularization (*r* = −0.33)	Lower extremity revascularization (*r* = −0.21)
Men	Baseline COT (*r* = 0.65)^‡^	Obesity (*r* = −0.60)^†^
	Baseline PWT (*r* = 0.64)^‡^	Abdominal obesity (*r* = −0.55)^†^
	Total volume of exercise (*r* = 0.53)^†^	Body mass index (*r* = −0.51)^†^
	Baseline 6-MWD (*r* = 0.50)^†^	Lower extremity revascularization (*r* = −0.46)^*∗*^

COT = claudication onset time, PWT = peak walking time, and 6-MWD = 6-minute walk distance.

Values are Pearson correlation coefficients.

^*∗*^ *p* < 0.05, ^†^*p* < 0.01, and ^‡^(*p* < 0.001).

**Table 4:** Multivariate regression models identifying predictors of the changes in ambulatory outcomes in women with symptomatic peripheral artery disease.

Dependent variables	Predictors	Regression coefficient	*R* ^2^	*p* values
ΔCOT (s)	Baseline COT	0.9280	0.27	0.007
	Intercept	−6.8782		
ΔPWT (s)	Baseline COT	0.4874	0.12	0.094
	Intercept	61.0904		

COT = claudication onset time; PWT = peak walking time.

**Table 5:** Multivariate regression models identifying predictors of the change in ambulatory outcomes in men with symptomatic peripheral artery disease.

Dependent variables	Predictors	Regression coefficient	*R* ^2^	*p* values
ΔCOT (s)	Baseline COT	0.6585	0.35	<0.01
	Obesity	−104.5220	0.07	0.10
	Intercept	106.9702		
ΔPWT (s)	Obesity	−284.2698	0.36	0.002
	Intercept	376.7143		

COT = claudication onset time; PWT = peak walking time.
